# Microwave Hydrothermal Synthesis of Terbium Ions Complexed with Porous Graphene for Effective Absorbent for Organic Dye

**DOI:** 10.1186/s11671-017-1962-7

**Published:** 2017-03-20

**Authors:** Keqin Chen, Hui Gao, Bowei Bai, Wenjing Liu, Xiaolong Li

**Affiliations:** 1 School of Physical Science and Technology, Key Laboratory for Magnetism and Magnetic Materials of the Ministry of Education, Lanzhou University, Lanzhou, 730000 People’s Republic of China; 2 Shanghai Synchrotron Radiation Facility, Shanghai Institute of Applied Physics, Chinese Academy of Sciences, Shanghai, 201204 People’s Republic of China

**Keywords:** Microwave, Absorbent, Rare earth ions, Graphene

## Abstract

A luminescent terbium ions/reduced graphene oxide complex (Tb-RGO) was successfully and rapidly synthesized by the microwave hydrothermal reaction via the interactions between terbium ions and the active oxygen functional groups of graphene oxide. The as-prepared material was porous stacked by multilayer graphene in all directions. Thus, the resulting product owed the high specific surface area, high adsorption capacity and ultra-fast adsorption rate. Combined with the characteristic photoluminescence derived from terbium ions, the material has potential applications in biosensing and environmental protection.

**Electronic supplementary material:**

The online version of this article (doi:10.1186/s11671-017-1962-7) contains supplementary material, which is available to authorized users.

## Background

Graphene with a hexagonal packed lattice structure is widely proposed to be a star material, boosted by its appealing properties, including superb mechanical property, high superficial area, eco-friendliness, etc. [1–4] As the “graphene gold rush” go on, tremendous niche applications have been developed, including sensors catalysts, energy conversion, and energy storage [5–7]. Especially in the functionalized graphene material field, sundry-modified materials emerge in an unending flow, deriving countless technologies and applications. It is versatile for the aforementioned functional application to produce reduced graphene oxide-based material, for its rich active oxygen functional groups can be ornamented before the reduced process [8, 9]. Generally, for these materials, the adsorption is a practical and common application and it has been reported that reduced graphene oxid with its derivative could effectively absorb manifold organic dyes, such as methylene blue (MB), Rhodamine B (RhB), through π-π stacking, hydrogen bonding, the electrostatic force, etc. [10, 11].

As another outstanding material, lanthanide ions are usually regarded as excellent dopants in nanoparticles and powders due to their characteristic features, including fine luminescence stability, high fluorescence quantum efficiency, low toxicity, and long luminescence lifetimes [12–15]. Among these elements, terbium (Tb) ions with the favorable green emissions have been the typical representative for doping into the matrix. Otherwise, terbium ions combined with graphene-based materials have been reported to be utilized for detecting mercury ions, monitoring hypochlorite, and enhancing the electrical conductivity, which indicated that wide applications were developed in the fields of optoelectronics and biosensing [16–18].

Based on logical speculation, amazing features will be generated for the compound-combined graphene and lanthanide ions. To obtain this imaginary material, seeking for the right preparation remains an obstacle. The composite’s composition, structure, and property are mainly affected by the method. Until now, there are many synthesis methods for graphene-based materials, including chemical vapor deposition, hydrothermal method, etc. [19, 20]. It is well known that the former method tends to require high synthesizing temperature while the latter will need relatively long time. Microwave hydrothermal method has been a popular method in synthesizing various nanoparticle-based materials with great superiority, such as high yield and contained cost. In the meantime, it owns the superior characteristic features containing fast heating, uniform heating throughout the whole material, mild reaction condition, and high energy utilization rate [21–23]. Based on these features, different properties of the product may be generated during microwave synthesis progress.

**Fig. 1:**
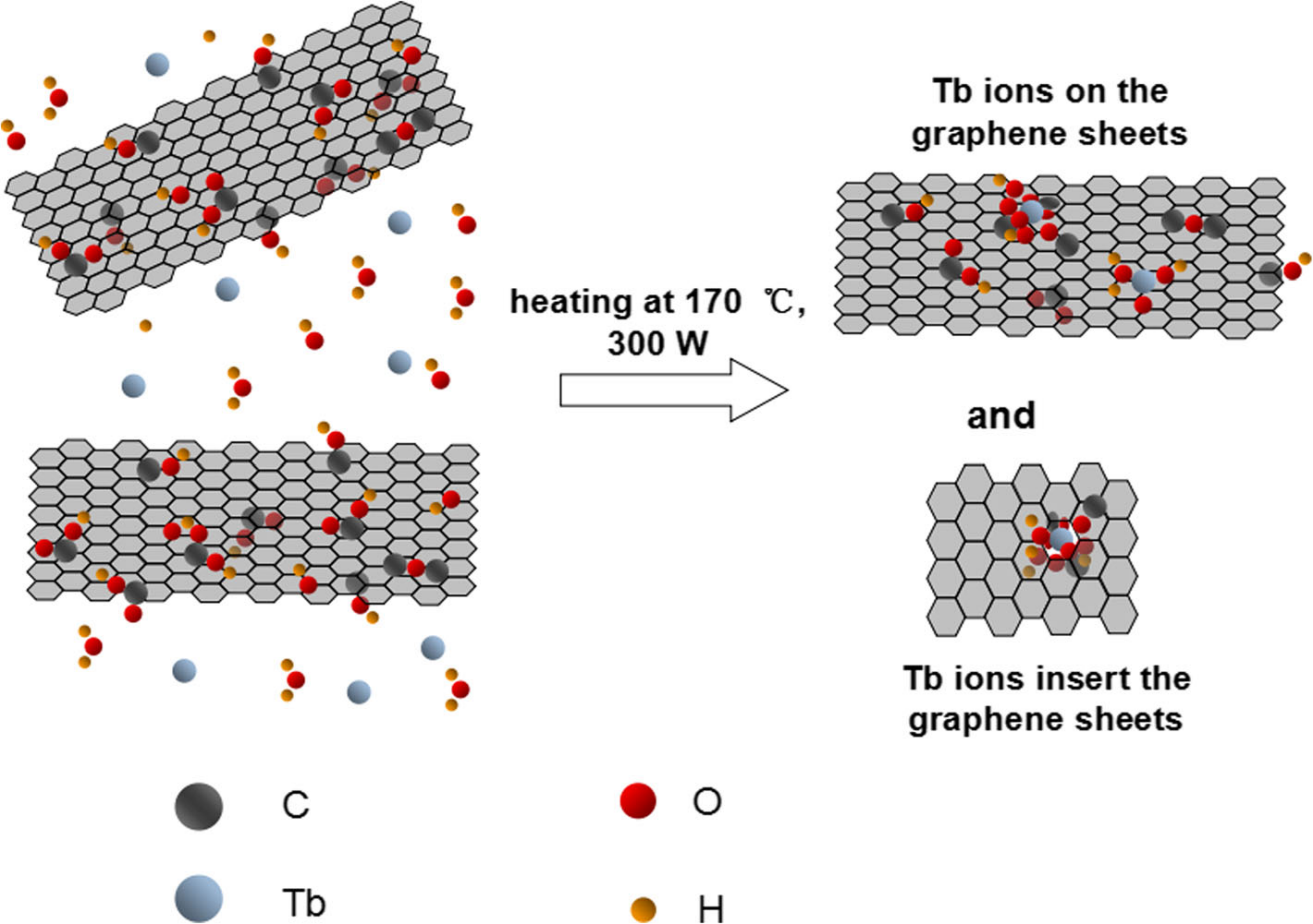
The proposal synthesis procedure of the graphene oxide modified with terbium ions

Herein ,Tb (III)/reduced graphene oxide was prepared, for the first time, through the microwave hydrothermal reaction as the enhanced adsorbent for organic dyes. Nevertheless, the compound owned a regular graphene lattice with relatively small size (3–5 nm), charming graphene quantum dots (GQDs) like photoluminescence emissions, induced by the interaction between Tb ions and oxygen functional groups on RGO. The fluorescence quenching test indicated that the adsorption process was accorded with the pseudo-first-order model and the as-prepared complex had ultrahigh removal rate. Hence, this compound we addressed can be applied in optoelectronics, quality adsorbence, and biosensing (Fig. 1).

## Methods

### Materials

Tb_4_O_7_ (99.9%) was purchased from Shanghai company. Terbium chloride was obtained by dissolving Tb_4_O_7_ in concentrated hydrochloric acid then matched to the concentration of 2 mg/mL.

### Preparation of Tb-RGO Hydrogels and Xerogels

Graphene oxide (GO) was prepared following the improved Hummers method [19]. For studying the time of reaching the adsorption equilibrium, considering the change of the color could be observed in time, we chose the concentration of 2.5 mg/mL for RhB to observe the adsorption process.

Twenty milliliters GO (3 mg/mL) and 2.4 mL TbCl_3_ (2 mg/mL) solutions were introduced into a Teflon-lined autoclave and heated at 170 °C for 20 min by microwave. The control group was performed like the above product except for replacing TbCl_3_ solution with distilled water. After being filtered, cylinder-like micro assemblies (hydrogel) were finally formed for Tb-complexed graphene and pristine graphene, respectively.

To preserve their original structures and get the xerogel, the as-prepared samples were subject to freeze-drying treatment. For short, the obtained Tb-complexed graphene and pristine graphene are called terbium ions/reduced graphene oxide complex (Tb-RGO) and reduced graphene oxide complex (RGO), respectively. The following experiments were designed for studying some best parameters and the results were applied for the best absorption experiment.

Considering the influence of the composition of materials for adsorption of RhB, eight kinds of Tb-RGO with different ratios of TbCl_3_ and RGO (1:2.5, 1:3.1, 1:4.1, 1:6.25, 1:12.5, 1:18.8, 1:25, 1:50) were prepared. For keeping the same amount of water, the concentrations of TbCl_3_ were separately 10, 8, 6, 4, 2, 1.5, 1, and 0.5 mg/mL. Then, the absorption experiment for the Tb-RGO hydrogels with different concentration was carried out.

To determine the influence of temperature, 1.0 g Tb-RGO hydrogels prepared under different temperatures (140, 150, 160, 170, and 180 °C) were mixed with 20 mL RhB dye solutions, respectively.

### Adsorption of Rhodamine B by Tb-RGO Hydrogels

To measure the adsorption capacity for as-prepared material, the swelling degree (SD) [24] related to the mass of the specific hydrogel (this material got by soaking in the deionized water with xerogel for 24 h) and xerogel, which were recorded as *m*_1_ and *m*_0_, respectively, was calculated using Eq. (1):

1 $$ \mathrm{S}\mathrm{D} = \left({m}_1\hbox{-}\ {m}_0\right)\ /{m}_0 $$

The mixtures mixing the as-prepared hydrogels and RhB dye solutions (15 and 2.5 mg/L) were placed at the room temperature. When it became colorless as time went on, it is then filtered. As we know, the adsorption procedure is reflected by the chroma, which is determined by the concentration of RhB. Due to the luminescence intensity of RhB being proportional to the concentration, the concentrations of RhB could be speculated through the relative luminescence intensity detected by fluorescence spectra. So, we could conjecture the concentration by the luminescence intensity to describe the adsorption progress. For RhB solutions, the concentration was determined by the intensity at 581 nm.

The removal efficiency of the dyes was calculated according to Eq.(2)

2 $$ \mathrm{Removal}\ \mathrm{efficiency}\ \left(\%\right)\kern1.23em \left({C}_0-{C}_{\mathrm{e}}\right)/\ {C}_0 \times 100 $$

Where *C*_0_ and *C*_e_ are the initial and equilibrium concentration of RhB solutions, respectively.

To measure the concentration of RhB correctly, the internal standard method was carried out. The standard RhB liquids of different concentration were detected by fluorescence spectrum.

### The Influence of Contact Time

To investigate the kinetics of the adsorption, 1.0 g Tb-RGO hydrogels with the best adsorption property were mixed with 15 mL RhB dye solutions, respectively. At predetermined time intervals (0, 10, 20, 30, 40, 50, 60 min), the concentrations of pollutant solutions were detected by the fluorescence spectra. The adsorption capacity as a function of time (*q*_t_) was calculated according to the following equation:

3 $$ {q}_{\mathrm{t}} = \left({C}_0 - {C}_{\mathrm{t}}\right) V/\  m $$

Where *C*_0_ is the concentration of the dye (mg/L) at 0 min. *C*_t_ is the concentration of the dye (mg/L) at *t* min. *V* is the volume of the solution and *m* is the mass of the xerogel. Two conventional kinetic models (pseudo-first-order and pseudo-second-order) as followed were used to simulate the experimental data.

4 $$ \mathrm{In}\left({q}_{\mathrm{e}}\hbox{-}\ {q}_{\mathrm{t}}\right) = \ln\ {q}_{\mathrm{e}}\hbox{-}\ {k}_1 t $$

5 $$ t/{q}_{\mathrm{t}}=1/\ {k}_2{q_{\mathrm{e}}}^2 + t\ /{q}_{\mathrm{e}} $$

Where *q*_e_ and *q*_t_ are the amounts of dyes adsorbed on the hydrogel (mg/g) at equilibrium time and *t* minutes, respectively. And *k*_1_ and *k*_2_ are the rate constants of pseudo-first-order and pseudo-second-order.

## Results and Discussion

**Fig. 2:**
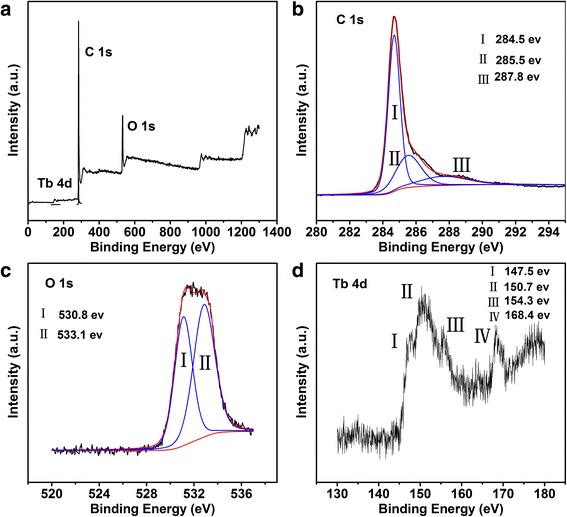
XPS survey spectrum of Tb-RGO (**a**). XPS high resolution spectra of C 1 s (**b**), O 1 s (**c**), and Tb 4d (**d**) core levels in Tb-RGO

To obtain the composition and structure of the as-prepared material, a series of characterization were carried out as followed description. The XPS survey spectra, indicating the valence state and chemical bonding of the Tb-RGO, shown in Fig. 2a. The analysis showed the distinctive presence of carbon (C) at ca. 284.6 eV, oxygen (O) at ca. 532.7 eV and Tb atoms at ca. 148 ~ 152 and 168 eV. There were three apparent spectral components at 284.5, 285.5, and 287.8 eV in the C 1 s peak (Fig. 2b). The main peak at a binding energy of 284.5 eV was assigned to sp^2^ hybridized C atoms in graphene, indicating the restoration of the C = C bonds after the hydrothermal reduction (~50%), whereas the other two peaks should be corresponded to sp^2^ C and sp^3^ C atoms bonded to O: epoxy/hydroxyls (C–O, 285.5 eV) and carbonyl (C = O, 287.8 eV) [25]. In addition, the O 1 s core level spectrum showed in Fig. 2c. The O 1 s peak could be divided into two peaks at 530.8 eV and 533.1 eV. As reported, the signal at lowest binding energy (529.0–531.1 eV) was assigned to the surface lattice oxygen and O–H, while the peak at higher energy (~533.0 eV) would involve various functional oxygen groups in RGO, such as C–OH, C = O, and O = C–OH [26, 27]. Through the Tb 4d core level spectrum shown in Fig. 2d, two regions were observed in this system suggesting the existence of different valence states [28, 29]. The 168.4 eV peak would be related to Tb^4+^ in Tb(III,IV) oxide, differing from Tb_4_O_7_ and Tb_2_O_3_. In addition, some peaks appeared in the low binding energy region due to the multiple splitting, indicating the interaction between Tb ions and RGO. Calculated by the formula of Tb/(Tb + C), in which the sign of atom means the peak area, the atomic concentration of Tb ions were 0.55%. The above XPS results indicated that the residual oxygen functionalities would be the bridge between the Tb ions and the RGO sheets. With the high ratio of O and C (O/C ~ 0.3), the formation of Tb–(O–C = O)_x_ and (H_2_O)_n_ · Tb–O–C had been proposed (Fig. 1).

The RGO and RGO-Tb had also been characterized using various physicochemical techniques (FTIR, Raman, SEM and HRTEM spectroscopy). Additional file 1: Figure S1(a) showed the typical features in the FTIR spectrum of RGO, such as the O–H stretching vibration (3429 cm^−1^), C = C from unoxidized sp^2^ C = C bands (1550-1620 cm^−1^), C–O vibration (1151 cm^−1^), and HO–C = O stretching vibration band (1718 cm^−1^). While in the FTIR spectrum of Tb-RGO, there were some differences, including arisen absorption band at 667 cm^−1^ of Tb–O, increasing band of C–O, subdued band of O−H. It can be inferred that Tb ions joined the molecules by replacing H atom of –OH, O = C–OH or breaking the C–O–C band. Raman spectra of the Tb-RGO and RGO were included in Additional file 1: Figure S1(b). In the Raman spectrum of RGO, the G peak was at 1590 cm^−1^ and D peak was at 1345 cm^−1^. The prominent D band was associated with the vacancies, grain boundaries, amorphous carbon species and the residual oxygen functionalities. The ratio of the D peak and the G peak slightly descended from 1.047 to 1.004, indicating the Tb-RGO had less introduced defects.

**Fig. 3:**
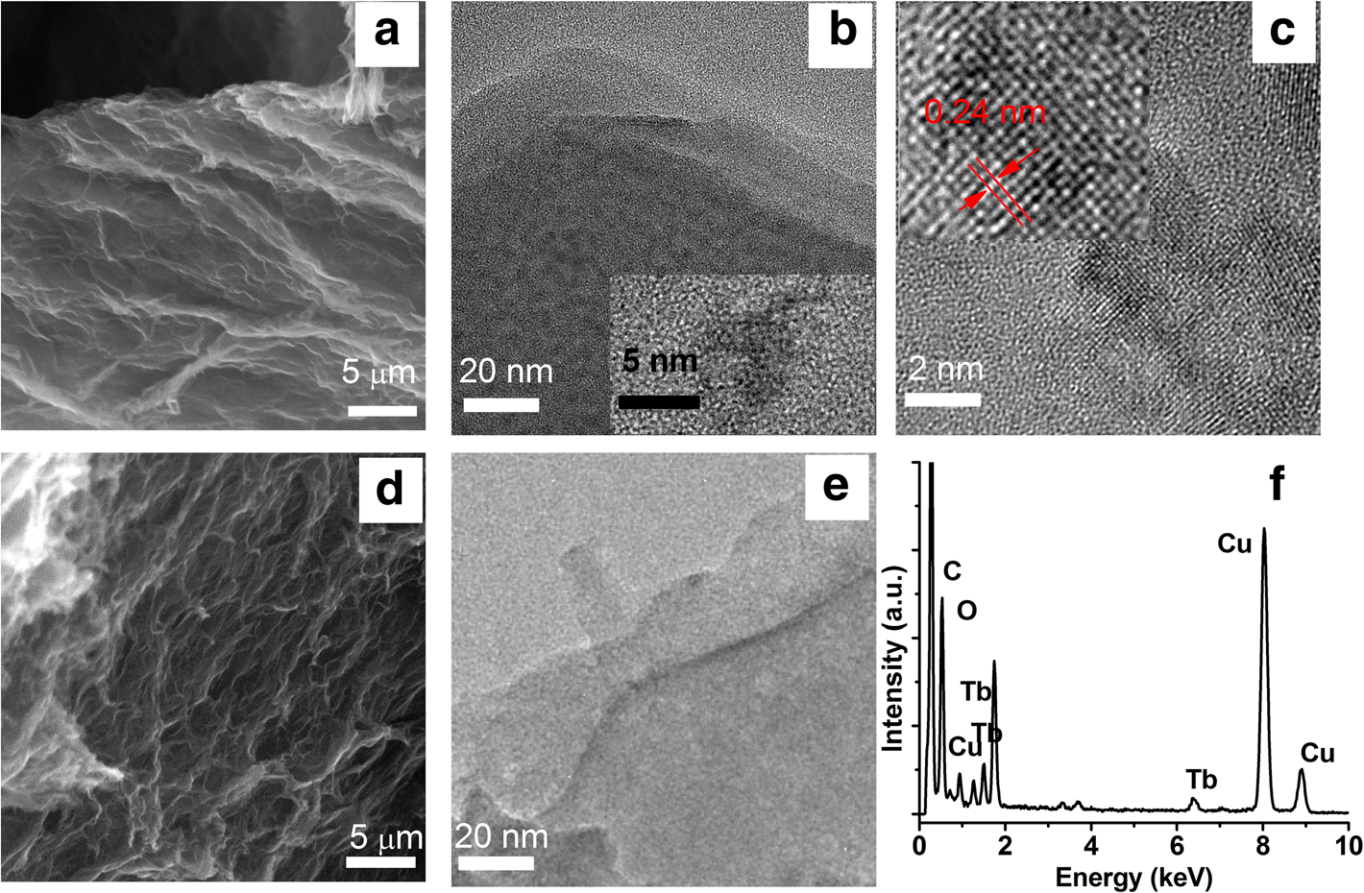
Electron microscopic characterization of reduced graphene oxide showing microstructural information. SEM images of Tb-RGO (**a**) and RGO (**d**). HRTEM images of Tb-RGO (**b**, **c**) and RGO (**e**). EDX analysis images of Tb-RGO (**f**)

The morphology of as-prepared materials shown in Fig. 3 was investigated by SEM and HRTEM, respectively. It could be observed that the structure of RGO were twisty and reunited together as the 3D porous between multilayer graphene sheets within the procedure, which was beneficial for adsorption process (shown in Fig. 3d). Compared with RGO, the sheets of Tb-RGO composite (shown in Fig. 3a) seemed more smooth and better-ordered, which was consistent with the analysis of the Raman results. What is more, a large number of dark dots distracted on the sheet of the composite uniformly while no dots but a very thin sheet appeared in RGO (shown in Fig. 3b, e). Through the high resolution TEM, it could be observed that the dots were formed with slightly fluctuant lattice and the interplanar crystal spacing was 0.24 nm (shown in Fig. 3c and inset graph). This was similar to the phenomenon of graphene quantum dots. The EDX image shown in Fig. 3f indicated the existence of Tb ions.

**Fig. 4:**
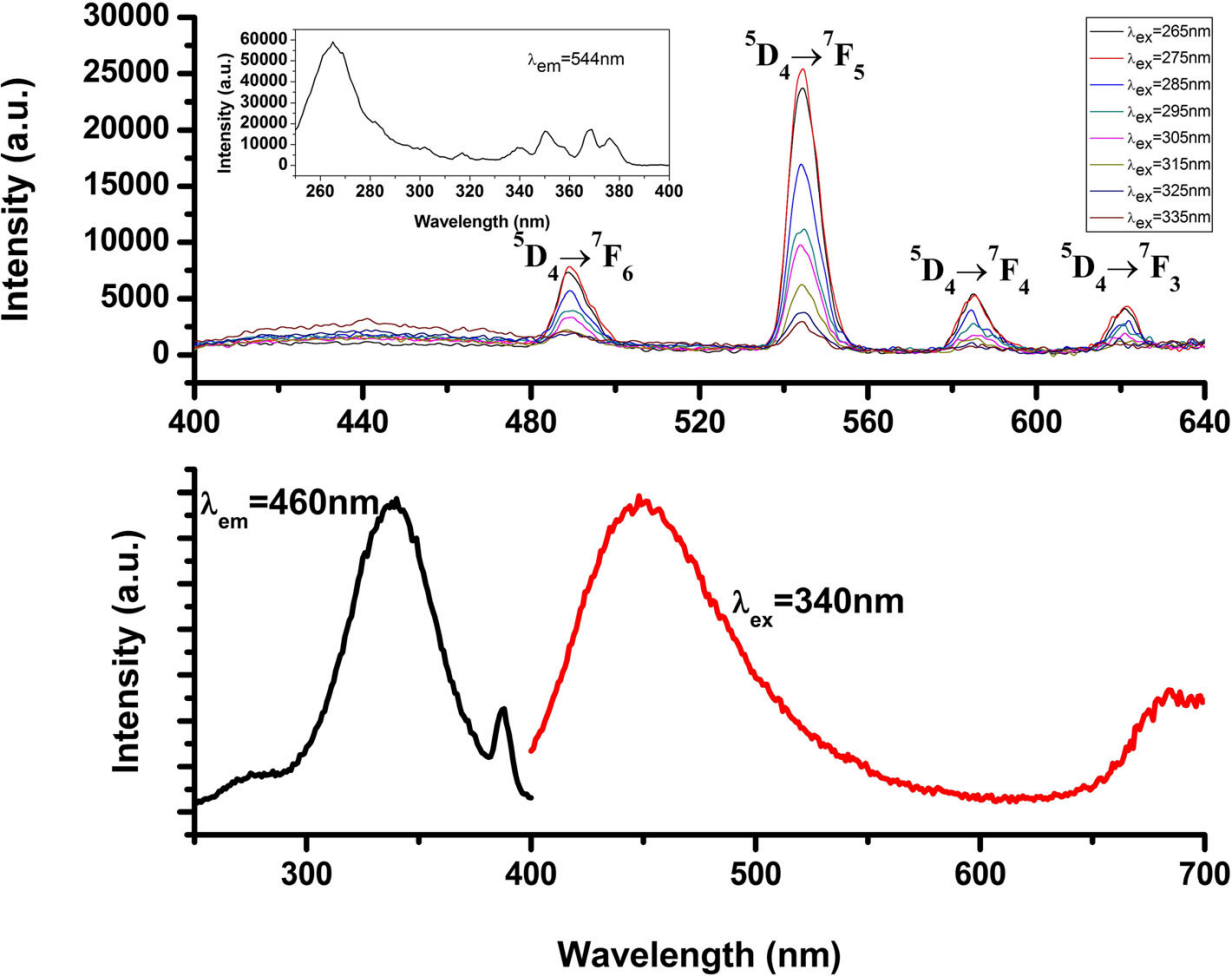
PL emission spectrum of Tb-RGO excited 265 to 335 nm and PL excitation spectrum of the complex (λ_m_ = 544 nm)

The corresponding photoluminescence (PL) emission spectra of Tb-RGO excited from 250 to 335 nm were shown in Fig. 4 (top). It was immediately clear that some relatively narrow PL peaks centered at 489, 544, 585, and 621 nm were observed, which actually were attributed to the ^5^D_4_–^7^F_J_ (*J* = 3, 4, 5, 6) transition of the Tb (III). It was also observed that the intensity of the PL emissions of Tb (III) decreased significantly when the excitation wavelength was above 265 nm, implying that wavelengths above 265 nm could not effectively excite the Tb (III) ions in this composite. The photoluminescence excitation (PLE) spectra of Tb-RGO were shown in the inset graph (monitored at 544 nm). Compared with pure Tb ions solution (Additional file 1: Figure S2), some linear peaks (from 300 to 380 nm) originated from the 4f-5d transition of Tb was faded, which could be attributed to the absorption of the matrix (shown in Fig. 4 (bottom)). What is more, the intensity of the excited peak (260–280 nm) improved, due to the enhanced transition rate after the incorporation of RGO. It is correlated with the adsorption spectra of GO in this region (3–5 eV), attributed to the interactions between the valence band and O 2p orbital, which indicated that the oxygen functionalities destroyed the symmetry of the π-π* system to widen the gap [30]. Hence, we proposed this model that the energy motivating Tb was from GO. And it could be observed that a broad peak appeared (400–550 nm), which attributed to the luminescence of graphene. Furthermore, it could be linked to the quantum dot effect. Likewise, the above results could be inferred that the bonding between Tb ions and RGO.

**Fig. 5:**
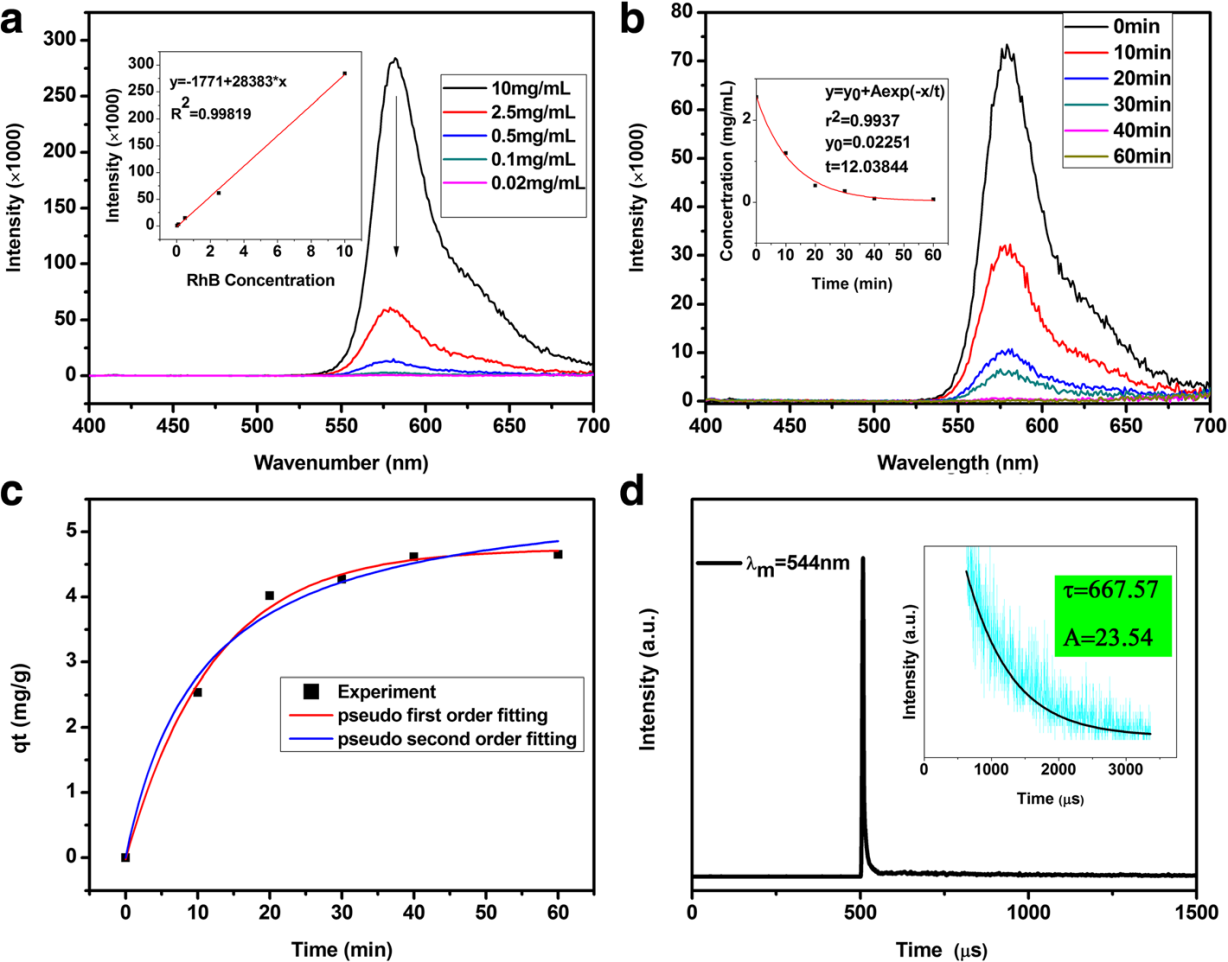
PL emission of RhB with different concentration (**a**). PL emission of RhB/RGO-Tb with different contact time (**b**). Fluorescence quenching kinetics fitting curves of Tb-RGO (**c**). Decay curve of Tb-RGO excited at 265 nm and monitored at 544 nm (**d**)

Except for PL and PLE analysis, the decay lifetime curve of Tb-RGO was shown in Fig. 5d for supporting the evidence the coupling between Tb ions and RGO. With excitation at 265 nm and recorded at the emission wavelength of 544 nm for Tb ion, the curve was roughly fitted to a single-exponential function, described with I(t) = A*exp(*-t*/*τ*). While the intensity of RGO was weak for measuring, which was attributed to the reduction progress of GO. As we all known, the decay life for composite with Tb were *τ* = 1 ~ 2000 μs according to different matrix, which supported more approach to transfer energy and descend the lifetime. The lifetime of the composite was τ = 667.57 μs, and the weighting parameter A was 23.54. The lifetime of Tb ions indicated the transition type is 4f-4f transition because the radiation probability to this parity-forbidden transition roughly is 10^3^ s^-1^ for crystals doped by rare earth ions (III) [31].

**Fig. 6:**
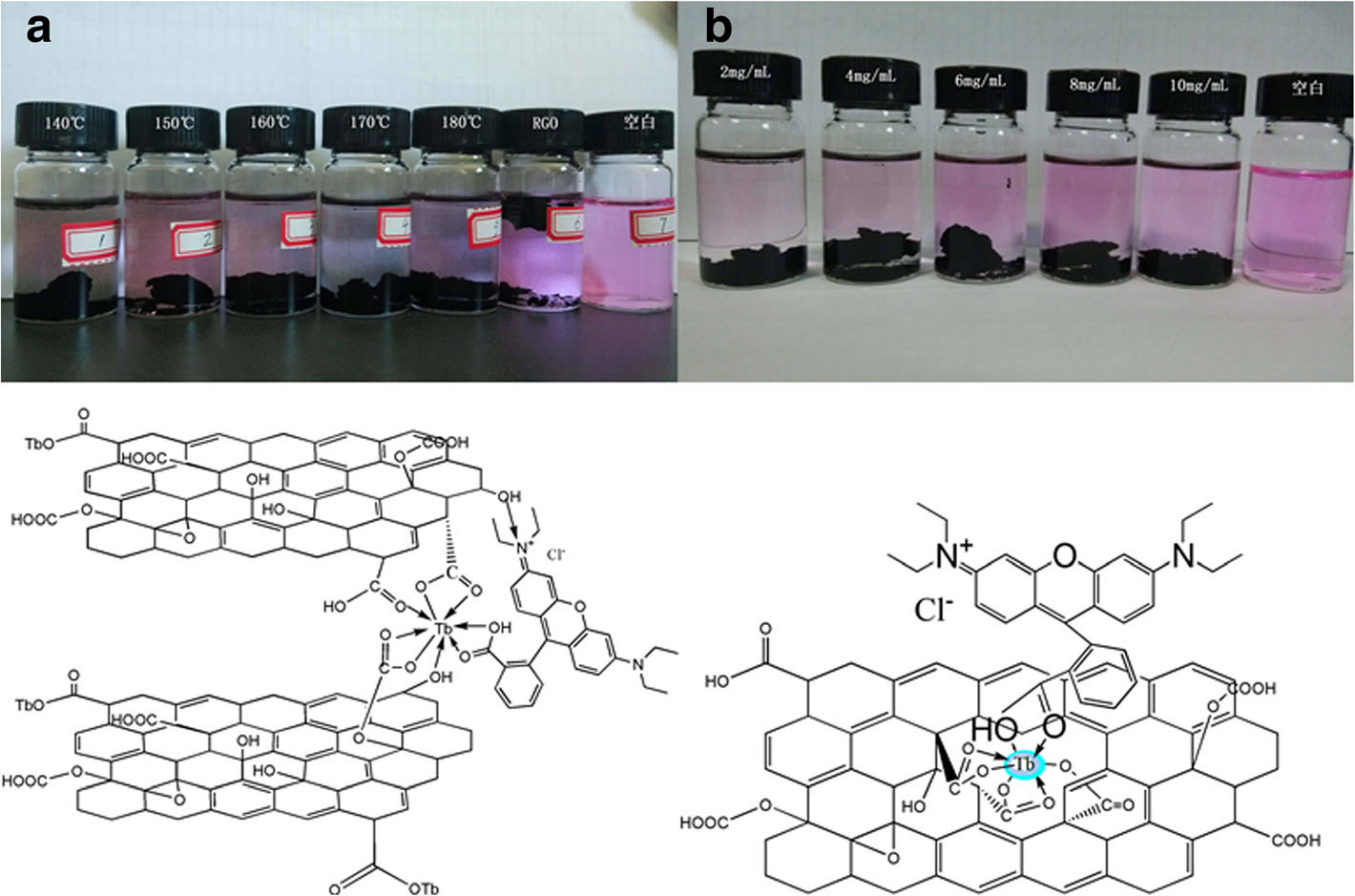
The adsorption test image under the different synthesis parameters (**a**) (**b**) and the proposal structure for the composite and RhB

Using Eq. (1), swelling degree of Tb-RGO xerogel was calculated to be about 80 mg/g, which was the average of five experiments. The date stated that the degree of crosslinking for the composite was high, which corresponding to the above morphology discuss. The result demonstrated that the Tb-RGO had strong adsorption potential. Here, we had used the organic fluorophore, Rhodamine B, to monitor the progress for its superb observability. As shown in Fig. 6a, b and Additional file 1: Figure S3, it was obvious that the color fades after mixing RGO or Tb-RGO hydrogel with Rhodamine B. The adsorption efficiency was related to the reaction temperature and the ratio of precursors (TbCl_3_ and GO), which was reflected by the concentration of TbCl_3_. It was clearly seen that the optimum reaction temperature and concentration of Tb to get the best removal efficiency for RhB are 170 °C and 2 mg/mL. So we used the material prepared under these conditions to study the adsorption property.

The adsorption progress could be explained that the concentration of luminophore was decrease due to the bonding between the hydrogel and RhB, which called fluorescence quenching. It took only 1 h for completely quenching of the fluorescence of Rhodamine B for Tb-RGO. While for RGO, a longer time (~8 h) was needed seen in Fig. 6b and Additional file 1: Figure S3. Thus, the absorption efficiency had been greatly improved by the introduction of Tb ions. It had been reported that the fluorescence quenching greatly depended on the electronic energy transfer and charge transfer. Thus, the introduction of Tb ions would inevitably tune the microstructure, electronic states of the RGO sheets and enhance the quenching ability for Rhodamine B. And we proposed two ways for Tb ions enhancing the adsorption based on its ability of central coordination shown at the bottom of Fig. 6.

Before research on the kinetics of adsorption process, the concentration of RhB should be measured correctly. The internal standard method curve was prepared (Fig. 4(top)) monitored at the summit of curve, indicating nearly proportional relation (the error might be caused by the background). From the kinetics of adsorption process (Fig. 4b), it was shown that the equilibrium time for RhB was about 60 min. Table 1 presents the kinetic parameters for the removal of dyes by the as-prepared hydrogels. For RhB, it was clearly seen that the correlation coefficient *R* ^2^ value of the pseudo-first-order model reached 0.99373. It was much higher than that of the pseudo-second-order model (0.98547). It was also found that the calculated adsorption capacity (q_e,cal_) obtained from the pseudo-first-order model, were 4.74120 mg/g. And obtained from the pseudo-second-order model were 5.70613 mg/g, which was close to the experimental adsorption capacity (q_e,exp_). The result indicated that, compared with the pseudo-second-order model, the pseudo-first-order model provided a better correlation for the adsorption of dyes on Tb-RGO. That was to say, the chemical reaction rate was directly proportional to the concentration of RhB.

**Table 1:** Kinetic parameters for the adsorption of RhB by Tb-RGO at the equilibrium time (60 min)

Q_e,exp_ (mg/g)	Pseudo-first-order			Pseudo-second-order		
	K1 (min^−1^)	Q_e,cal_ (mg/g)	*R* ^2^	K2 (g/(mg*min))	Q_e,cal_ (mg/g)	*R* ^2^
5.0011	0.08311	4.74120	0.99373	0.01670	5.70613	0.98547

## Conclusions

In conclusion, we found a facile time-saving approach to synthesize Tb-RGO hydrogels at relatively low temperature (170 °C). The as-obtained hydrogels exhibited the high surface area with a porous structure. And the Tb-RGO hydrogels were applied as adsorbents for RhB from the aqueous solutions. The results indicated that there were high removal efficiencies for RhB due to the strong π-π stacking and special structure. The equilibrium time of adsorption was 60 min for RhB and the solution could be decolorized to nearly colorless. The concentration of Tb and the reaction temperature were further investigated for removal process. And the kinetics of removal process was researched. The results demonstrated that Tb-RGO hydrogels would have great potential for the practical application in adsorption material.

## Additional file

Additional file 1: Microwave hydrothermal synthesis of terbium ions complexed with porous graphene for effective absorbent for organic dye. (DOCX 2650 kb)

## Abbreviations

GO: Graphene oxide
MB: Methylene blue
RhB: Rhodamine B
Tb: Terbium
Tb-RGO: Terbium ions/reduced graphene oxide complex

## References

[1] Jahan M, Bao Q, Loh KP (2012). Electrocatalytically active graphene–porphyrin MOF composite for oxygen reduction reaction. J Am Chem Soc.

[2] Loh KP, Bao Q, Ang PK, Yang J (2010). The chemistry of graphene. J Mater Chem.

[3] Si Y, Samulski ET (2008). Exfoliated graphene separated by platinum nanoparticles. Chem Mater.

[4] Lightcap IV, Kosel TH, Kamat PV (2010). Anchoring semiconductor and metal nanoparticles on a two-dimensional catalyst mat. Storing and shuttling electrons with reduced graphene oxide. Nano Lett.

[5] Huang X, Qi X, Boey F, Zhang H (2012). Graphene-based composites. Chem Soc Rev.

[6] Novoselov KS, Falko VI, Colombo L, Gellert PR, Schwab MG, Kim K (2012). A roadmap for graphene. Nature.

[7] Young RJ, Kinloch IA, Gong L, Novoselov KS (2012). The mechanics of graphene nanocomposites: a review. Compos Sci Technol.

[8] Eda G, Fanchini G, Chhowalla M (2008). Large-area ultrathin films of reduced graphene oxide as a transparent and flexible electronic material. Nat Nano.

[9] Compton OC, Nguyen ST (2010). Graphene oxide, highly reduced graphene oxide, and graphene: versatile building blocks for carbon-based materials. Small.

[10] Wang D, Gao H, Roze E, Qu K, Liu W, Shao Y, Xin S, Wang Y (2013). Synthesis and photoluminescence of three-dimensional europium-complexed graphene macroassembly. J Mater Chem C.

[11] Wang X, Liu Z, Ye X, Hu K, Zhong H, Yu J, Jin M, Guo Z (2014). A facile one-step approach to functionalized graphene oxide-based hydrogels used as effective adsorbents toward anionic dyes. Appl Surf Sci.

[12] Latva M, Takalo H, Mukkala V-M, Matachescu C, Rodríguez-Ubis JC, Kankare J (1997). Correlation between the lowest triplet state energy level of the ligand and lanthanide (III) luminescence quantum yield. J Lumin.

[13] Fan X, Shang K, Sun B, Chen L, Ai S (2014). Decoration of surface-carboxylated graphene oxide with luminescent Sm3 + -complexes. J Mater Sci.

[14] Li P, Wang Y, Li H, Calzaferri G (2014). Luminescence enhancement after adding stoppers to europium (III) nanozeolite L. Angew Chem Int Ed.

[15] Chen Y, Chi Y, Wen H, Lu Z (2007). Sensitized luminescent terbium nanoparticles: preparation and time-resolved fluorescence assay for DNA. Anal Chem.

[16] Li Q-P, Yan B (2012). Multi-walled carbon nanotube-based ternary rare earth (Eu 3+, Tb 3+) hybrid materials with organically modified silica–oxygen bridge. J Colloid Interface Sci.

[17] Liao L, Peng H, Liu Z (2014). Chemistry makes graphene beyond graphene. J Am Chem Soc.

[18] Zondlo SC, Gao F, Zondlo NJ (2010). Design of an encodable tyrosine kinase-inducible domain: detection of tyrosine kinase activity by terbium luminescence. J Am Chem Soc.

[19] Hummers WS, Offeman RE (1958). Preparation of graphitic oxide. J Am Chem Soc.

[20] Kim KS, Zhao Y, Jang H, Lee SY, Kim JM, Kim KS, Ahn J-H, Kim P, Choi J-Y, Hong BH (2009). Large-scale pattern growth of graphene films for stretchable transparent electrodes. Nature.

[21] Lidström P, Tierney J, Wathey B, Westman J (2001). Microwave assisted organic synthesis—a review. Tetrahedron.

[22] Gerbec JA, Magana D, Washington A, Strouse GF (2005). Microwave-enhanced reaction rates for nanoparticle synthesis. J Am Chem Soc.

[23] Bensebaa F, Patrito N, Le Page Y, L'Ecuyer P, Wang D (2004). Tunable platinum–ruthenium nanoparticle properties using microwave synthesis. J Mater Chem.

[24] Panic VV, Madzarevic ZP, Volkov-Husovic T, Velickovic SJ (2013). Poly (methacrylic acid) based hydrogels as sorbents for removal of cationic dye basic yellow 28: kinetics, equilibrium study and image analysis. Chem Eng J.

[25] Belaya SV, Bakovets VV, Boronin AI, Koshcheev SV, Lobzareva MN, Korolkov IV, Stabnikov PA (2014). Terbium oxide films grown by chemical vapor deposition from terbium(III) dipivaloylmethanate. Inorg Mater.

[26] Xu H, Li G, Li J, Chen C, Ren X (2016). Interaction of Th(IV) with graphene oxides: Batch experiments, XPS investigation, and modeling. J Mol Liq.

[27] Compton OC, Jain B, Dikin DA, Abouimrane A, Amine K, Nguyen ST (2011). Chemically active reduced graphene oxide with tunable C/O ratios. ACS Nano.

[28] Guodong F, Changgen F, Zhao Z (2007). Surface and texture properties of Tb-doped ceria-zirconia solid solution prepared by sol-gel method. J Rare Earth.

[29] Dai H, Ng C, Au C (2001). SrCl 2-promoted REO x (RE = Ce, Pr, Tb) catalysts for the selective oxidation of ethane: a study on performance and defect structures for ethene formation. J Catal.

[30] Yeh T-F, Chan F-F, Hsieh C-T, Teng H (2011). Graphite oxide with different oxygenated levels for hydrogen and oxygen production from water under illumination: the band positions of graphite oxide. J Phys Chem C.

[31] Klink SI, Grave L, Reinhoudt DN, van Veggel FCJM, Werts MHV, Geurts FAJ, Hofstraat JW (2000). A systematic study of the photophysical processes in polydentate triphenylene-functionalized Eu3+, Tb3+, Nd3+, Yb3+, and Er3+ complexes. J Phys Chem A.

