# Supplementary material for: Microwave Hydrothermal Synthesis of Terbium Ions Complexed with Porous Graphene for Effective Absorbent for Organic Dye
Source: Nanoscale Res Lett. 2017 Mar 20;12:204. doi: 10.1186/s11671-017-1962-7 (PMC5359191; doi:10.1186/s11671-017-1962-7)
Supplement: Additional file 1: — Microwave hydrothermal synthesis of terbium ions complexed with porous graphene for effective absorbent for organic dye. (DOCX 2650 kb) [file 11671_2017_1962_MOESM1_ESM.docx]

**Microwave hydrothermal synthesis of terbium ions complexed with porous graphene for effective absorbent for organic dye**

**Keqin Chen ^a,^** ^‡^**, Hui Gao ^a,^** ^‡^ **^,*^, Bowei Bai ^a^, Wenjing Liu ^a^, Xiaolong Li ^b*^**

a School of Physical Science and Technology, Key Laboratory for Magnetism and Magnetic Materials of Ministry of Education, Lanzhou University, Lanzhou 730000, P.R. China

b Shanghai Synchrotron Radiation Facility, Shanghai Institute of Applied Physics, Chinese Academy of Sciences, Shanghai 201204, P. R. China

***Corresponding authors:** [hope@lzu.edu.cn](mailto:wyh@lzu.edu.cn) (H. Gao)

lixiaolong@sinap.ac.cn (X.L. Li)

Tel.: +86-18993181143, Fax: +86-931-8913554

‡ These authors contributed equally to the article.


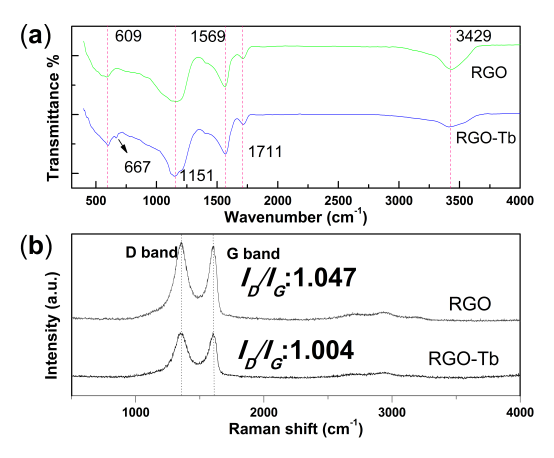


**Fig. S1** the FT-IR (a) and Raman (b) spectra of RGO and Tb-RGO.


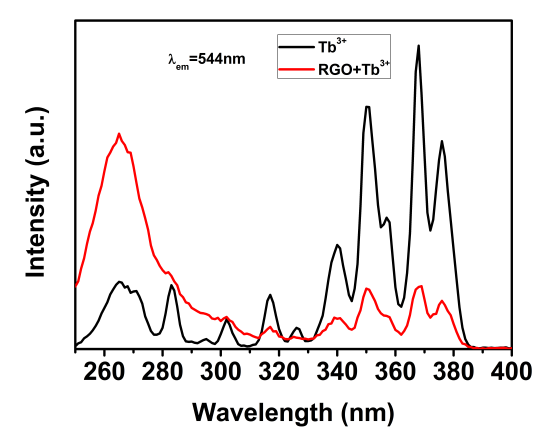


**Fig. S2** the photoluminescence excitation spectra of TbCl3 and Tb-RGO composite monitored at 544nm.


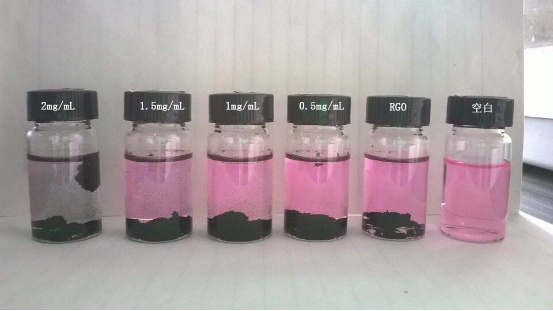


**Fig. S3** the adsorption test image under different synthesis parameters.
